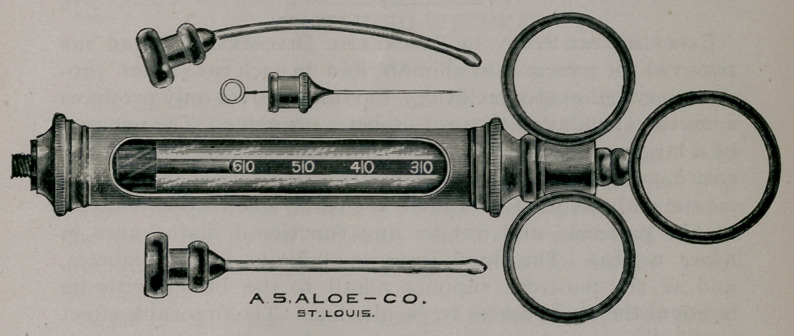# An Improved Syringe for Infiltration Anæsthesia

**Published:** 1896-03

**Authors:** 


### AN IMPROVED SYRINGE FOR INFILTRATION ANÆSTHESIA.

During the past year, I have been making large use of Schleich's method of producing local anæsthesia by infiltrating tissues with innocuous solutions of salt, morphine, cocaine, etc., the anæsthetic effect resulting from the method of applying the fluid rather than from the drug or drugs employed in it. It has been a source of great satisfaction to me and the patients on whom it was used. The minuteness of strength of drug employed removes every element of danger from that source, even though a large quantity of the solution be injected and the completeness and promptness of the effects are advantages readily evident.

But I have met with one objection that has restricted the use of the method to a marked degree. It is the fact that, no matter how easily and satisfactorily it may be employed in the superficial structures, where there are large vessels in danger of being punctured with the hypodermic needle when one is injecting in the depths of a wound in the neighborhood of large arteries or veins, as in enucleating bubo-glands immediately above the femoral vessels, etc., the likelihood of running the needle into one of them and producing disastrous results is not a fancied one.

By means of the needles represented in the cut, I have been enabled to do away with this difficulty. They are blunt-pointed and made of German silver, so that, though of sufficient stiffness to be thrust into the connective tissues of a wound after the skin has been severed, they would not injure a blood vessel if pushed against one.

The anæsthesia is begun, therefore, with the sharp steel needle and continued with either of the two silver ones. The choice between the latter depends on whether a curved or straight needle is more conveniently used.

The advantages offered by this improvement have shown themselves to be eminently practical and serviceable, and, in my estimation, will advance the scope and usefulness of the method to a great degree. This instrument was made for me by the A. S. Aloe Co. of St. Louis, Mo.

---

EXPERIMENTAL STUDY OF PANCREATIC DIABETES.—Chabad has removed the pancreas in animals, and in each case it has produced acute diabetes mellitus. Partial removal only produces symptoms similar to those of diabetes insipidus. The removal of a larger portion causes slight diabetes mellitus, and of the entire pancreas the acute form. He concludes that the pancreatic diabetes thus produced is entirely due to the removal of the pancreas and not to any functional disturbance in other organs. The blood loses its alkilinity in this diabetes, and as the pancreas supplies alkali to the blood, with its removal the blood ceases to be alkaline. The favorable effect of alkalies in diabetes can thus be explained by these experiments on animals. They have fully established the fact of the existence of pancreatic diabetes.—*Gazz. degli Osp. e Dlin.*

## Figures and Tables

**Figure f1:**